# Comprehensive Transcriptomic Analysis and Biomarker Prioritization of Hydroxyprogesterone in Breast Cancer

**DOI:** 10.3390/cimb48010108

**Published:** 2026-01-20

**Authors:** Abdallah Rafi, Şükrü Tüzmen, Osman Uğur Sezerman, Fikret Dirilenoğlu

**Affiliations:** 1 Faculty of Medicine, Near East University, North Cyprus Via Mersin 10, 99138 Nicosia, Türkiyefikret.dirilenoglu@neu.edu.tr (F.D.); 2 Faculty of Dentistry, Eastern Mediterranean University, North Cyprus Via Mersin 10, 99628 Famagusta, Türkiye; 3 GenBiomics R&D, Eastern Mediterranean University, Techno Park, North Cyprus Via Mersin 10, 99628 Famagusta, Türkiye; 4 Faculty of Medicine, Acıbadem University, 34753 Istanbul, Türkiye; sezermanu@gmail.com

**Keywords:** omics, transcriptomics, breast cancer, hydroxyprogesterone (HP), biomarker discovery

## Abstract

Hydroxyprogesterone (HP) is a synthetic progestogen widely used in obstetric care, and its potential influence on breast cancer biology has become an emerging area of interest. Despite its clinical use, the molecular mechanisms by which HP affects tumor tissue remain insufficiently explored. In this study, transcriptomic profiling was performed to investigate gene expression changes associated with HP in operable breast cancer. Pre-operative 17α-HP caproate (17-OHPC) exposure was associated, in normal adjacent tissue (NAT), with activation of steroid-hormone and lipid/xenobiotic-metabolism programs and crosstalk to phosphoinositide 3-kinase (PI3K)–Akt and nuclear factor kappa B (NF-κB). In NAT, these pathways showed the largest absolute log_2_ fold-change (|log_2_FC|); significance is reported as false discovery rate (FDR) throughout (e.g., FKBP5↑ with HP). In tumor tissue, the dominant signal reflected tight-junction/apical-junction and extracellular matrix (ECM)-receptor remodeling (e.g., CLDN4↑). We prioritized FKBP5 (HP pharmacodynamics) and CLDN4 (tumor baseline) as the main candidates; TSPO and SGK1 are reported as exploratory. This discovery-level, hypothesis-generating analysis nominates candidate biomarkers and pathway signals for prioritization and evaluation in independent datasets and future studies. These findings provide mechanistic insight into HP’s molecular effects in breast cancer and suggest potential applications in biomarker perioperative management.

## 1. Introduction

Breast cancer remains the most prevalent malignancy in women worldwide and a leading cause of cancer-related mortality. In 2020 alone, 2.3 million new cases and approximately 685,000 deaths were reported, accounting for 16% of all female cancer deaths [1,2,3]. While incidence is higher in developed regions, mortality disproportionately affects transitioning countries due to delayed diagnosis and limited access to effective therapies [4,5]. Global projections estimate that annual breast cancer cases may surpass 3 million by 2040, highlighting the urgent need for enhanced prevention, early detection, and biomarker-driven treatment strategies [3].

Hormonal signaling plays a critical role in breast cancer pathogenesis. Estrogen receptors (ERα/ERβ) and progesterone receptors (PR-A/PR-B) regulate gene transcription, cell proliferation, and tumor progression. Estrogen primarily drives tumorigenesis through both genomic and non-genomic pathways, while progesterone exerts complex effects, sometimes promoting survival and proliferation [6,7]. Hydroxyprogesterone (HP), a bioactive PR ligand widely used in obstetric care, has emerged as a compound of interest in breast cancer biology. Experimental evidence suggests that HP and related progestogens modulate tumor metabolism, attenuate glycolysis, and influence anti-inflammatory signaling pathways, potentially impacting therapy response and resistance mechanisms [8,9,10]. Recent advances in precision oncology emphasize the utility of transcriptomics in unraveling hormone-driven tumor biology. Transcriptome profiling captures dynamic gene expression, alternative splicing, and non-coding RNA activity, providing real-time molecular insights beyond static genomic mutations [11]. Clinically, transcriptomic assays like Oncotype DX and MammaPrint have demonstrated utility in risk stratification and guiding endocrine therapy in ER-positive breast cancer [12,13]. Throughout, “HP+” denotes pre-operative 17-OHPC exposure and “HP−” denotes no exposure. When integrated with co-expression network analysis, transcriptomics facilitates biomarker discovery by connecting expression changes to functional protein targets with translational relevance [14].

**Figure 1:**
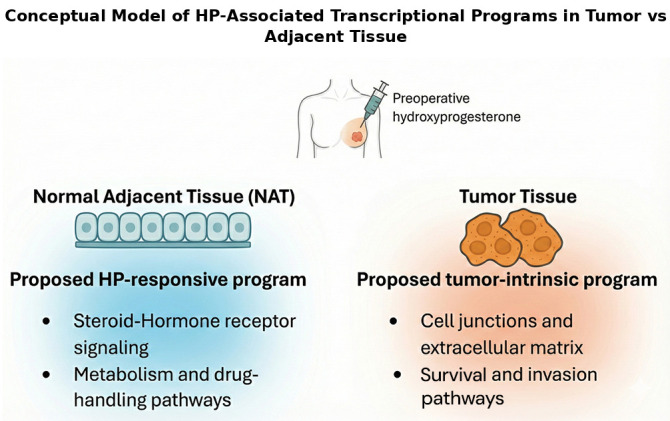
Conceptual model of HP-associated transcriptional programs in tumor vs. adjacent tissue. Credit: Sketched by the author in Samsung Notes (v4.4.30.91); redrawn in Microsoft Word (Microsoft 365, v2511); vector-cleaned with ChatGPT (version 5.1, OpenAI OpCo, LLC, 1455 3rd Street, San Francisco, CA 94158, USA; web version, accessed 15 November 2025) from the original author sketch.

Short-window pre-operative exposure to 17-OHPC may differentially affect normal adjacent tissue (NAT) and tumor; therefore, we profiled matched tumor/NAT transcriptomes to map exposure-linked signals. Our goals were (i) to delineate HP-associated transcriptional programs in each tissue and (ii) to assess whether these programs align with two complementary axes—steroid-hormone/lipid–drug-metabolism in NAT versus tight-junction/ECM remodeling in tumor. As an exploratory, hypothesis-generating analysis, we prioritized candidate biomarkers using a predefined rubric and summarized module-level trends with Weighted Gene Co-expression Network Analysis (WGCNA) and pathway enrichment. This conceptual dual-axis model is outlined in Figure 1. Accordingly, we framed the results as discovery-level and hypothesis-generating, aimed at biomarker nomination and pathway-level insight to motivate follow-up studies. This study re-analyzes a publicly available RNA-seq dataset and extends the original work by emphasizing tumor–NAT compartment specificity and integrating pathway/co-expression analyses to prioritize candidate biomarkers [15].

## 2. Materials and Methods

### 2.1. Study Design and Overview

This study was designed to explore the molecular effects of HP on operable breast cancer using an integrative bioinformatics approach. The primary goal was to identify transcriptomic alterations induced by HP treatment and to prioritize potential biomarkers with functional relevance. The research utilized publicly available RNA-seq data from the Sequence Read Archive (SRA) project ERP135222, which contains HP-exposed and non-exposed samples from tumor and NAT collected from patients exposed or unexposed to HP prior to surgery [16].

To capture the complexity of HP’s effects, we categorized samples into four biologically relevant groups: Tumor HP+, Tumor HP−, NAT HP+, and NAT HP−. This scheme enables within-tissue HP contrasts (Tumor HP+ vs. Tumor HP−; NAT HP+ vs. NAT HP−) and cross-tissue comparisons (Tumor vs. NAT). Table 1 summarizes patient/sample counts. Based on the source article’s clinical summary, HP-exposed patients (*n* = 18; 58.6 ± 2.8 y) were evenly distributed across ER+PR±HER2−, HER2+, and ER−PR−HER2− groups (6/18 each; 33.3%). Unexposed patients (*n* = 13; 58.5 ± 9.7 y) included 4/13 (30.8%) ER+PR±HER2−, 8/13 (61.5%) HER2+ (of which 6 ER+HER2+ and 2 ER−HER2+), and 1/13 (7.7%) ER−PR−HER2−.

**Figure 2:**
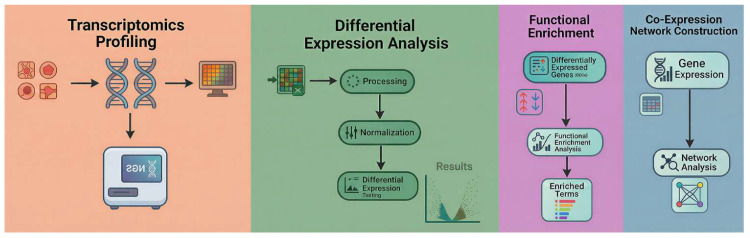
Integrated workflow combining transcriptome profiling, differential expression, functional enrichment, and network analysis. Credit: Sketched by the author in Samsung Notes (v4.4.30.91); redrawn in Microsoft Word (Microsoft 365, v2511); vector-cleaned with ChatGPT (version 5.1, OpenAI OpCo, LLC, 1455 3rd Street, San Francisco, CA 94158, USA; web version, accessed 15 September 2025) from the original author sketch.

The overall workflow, as shown in Figure 2, combined transcriptome profiling, differential expression analysis, functional enrichment, and co-expression network construction, thereby providing a multi-scale evaluation of HP’s influence on breast cancer biology.

### 2.2. Dataset Description and Sample Grouping

The dataset included 31 women diagnosed with operable breast cancer. All patient recruitment, sample collection, RNA extraction, library preparation, and sequencing were performed in the original study; the present manuscript analyzes the deposited sequencing data and available metadata. Among these patients, 18 received a single intramuscular dose of 500 mg HP within 15 days before surgery, while 13 underwent surgery without hormonal exposure. Multiple core biopsies were collected, encompassing pre-surgical and post-resection specimens for both tumor and NAT [16]. In total, 31 tumor samples and 10 NAT samples were analyzed. Laboratory procedures and reagents (including the reported RNA purification kit) are described in the original study documentation and associated dataset records [17]. Per-sample clinical metadata were incomplete and non-uniform (including age, stage, survival outcomes, and ER/PR/HER2 status); thus, analyses were not covariate-adjusted, and subtype-level inference was treated as exploratory.

### 2.3. Data Preprocessing

High-quality transcriptomic data is critical for reliable downstream analyses. Raw sequencing data, initially in SRA formats, were retrieved from the SRA and converted to standard paired-end FASTQ files using the SRA Toolkit (fasterq-dump, version 3.2.1) [18]. Conversion integrity was verified by confirming read pair consistency and file sizes.

Next, adapter trimming and quality filtering were conducted using Trim Galore, which incorporates Cutadapt (version 5.2) and FastQC (version 0.12.1) [19,20]. This step removed adapter sequences, low-quality bases (Phred < 20), and ambiguous poly-N regions. Post-trimming quality checks using FastQC confirmed that retained reads achieved mean Phred scores ≥30 and preserved >90% of their original base content.

### 2.4. Alignment and Quantification

The filtered reads were aligned to the human reference genome GRCh38 using HISAT2 (version 2.2.1) [21], a splice-aware aligner that efficiently handles mammalian transcriptomes. Reference indices were generated from the GRCh38 FASTA sequence and its corresponding GTF annotation file to support precise, exon-aware mapping. Alignment quality metrics, including overall mapping rates and the percentage of uniquely aligned reads, were reviewed to ensure consistency across samples. Gene-level quantification was performed using featureCounts (version 2.0.6) [22], which generated a matrix of raw read counts for each gene in each sample.

### 2.5. Differential Expression Analysis

Differential expression analysis was performed to identify genes modulated by HP exposure and tissue type. Raw count matrices were normalized using DESeq2 (version 1.49.4) [23] and edgeR (version 4.6.3) [24], which apply the median-of-ratios and TMM (trimmed mean of M-values) methods, respectively.

Comparisons were performed between tumor tissues with and without HP exposure, between NAT with and without HP exposure, and between tumor and NAT under both treatment and untreated conditions. This approach enabled the identification of genes that were specifically influenced by hormonal exposure and those that were characteristic of the tumor environment itself.

Low-expression genes (fewer than 10 counts in ≥3 samples) were filtered out. Genes meeting an adjusted *p*-value (FDR) < 0.05 and |log_2_ fold change| > 1 were considered significantly differentially expressed (DEGs). The final DEG lists represented high-confidence transcriptional changes for downstream pathway and network analyses [23,24]. An interaction design was considered in exploratory analyses to examine context-dependent HP effects. Given the small per-group sample sizes and incomplete per-sample subtype annotation, subtype-stratified models and higher-order interaction testing were not emphasized and are treated as exploratory.

### 2.6. Functional Enrichment Analysis

To interpret the biological significance of the DEGs, both Overrepresentation Analysis (ORA) and Gene Set Enrichment Analysis (GSEA) were performed. Gene Ontology (GO) terms across biological processes (BP), molecular functions (MF), and cellular components (CC), as well as KEGG pathways, were assessed for enrichment using the clusterProfiler R package (v4.18.0) [25]. ORA identified overrepresented pathways among discrete DEG lists, while GSEA detected coordinated shifts in predefined gene sets across the entire expression ranking [26]. For GSEA, significance was defined as FDR q < 0.25 (primary), with q < 0.05 providing a systems-level view of hormone-driven transcriptomic alterations.

### 2.7. Co-Expression Network Construction (WGCNA)

WGCNA for downstream pathway and network analyses [27] was applied to identify modules of co-expressed genes associated with tissue phenotype and HP treatment. Variance-stabilized counts from DESeq2 served as the input matrix, and low-variance genes were filtered out to reduce noise.

A soft-thresholding power (β) was selected based on the scale-free topology criterion to produce a biologically meaningful network. Modules were detected using hierarchical clustering and the dynamic tree-cut algorithm, and module eigengenes were correlated with clinical traits. Modules showing significant associations with HP exposure or tumor phenotype were selected for hub gene identification.

### 2.8. Transcriptome-Based Biomarker Prioritization

Candidate biomarkers were prioritized by integrating differential expression, network centrality, and biological validation. High-confidence DEGs that also functioned as hub genes within significant co-expression modules were assessed in curated databases, including DisGeNET [28], Human Protein Atlas [29], and GeneCards [30], to evaluate their known functional roles and clinical relevance. Candidate selection criteria: genes were prioritized by (i) DE significance in prespecified contrasts, (ii) biological plausibility for progestin signaling, (iii) concordance across GO/KEGG/GSEA, and (iv) WGCNA module membership (kME) in HP-treated or tumor-enriched modules. 

Genes that were consistently supported across these layers were considered top-priority transcriptomic biomarkers. This multi-tiered strategy ensured that shortlisted biomarkers were both statistically robust and clinically meaningful, providing a foundation for downstream studies.

## 3. Results

### 3.1. Quality Control and Read Statistics

Before downstream analyses, we performed RNA-seq QC to confirm sequencing depth and normalization across tumor and NAT. Per-sample gene-assigned reads showed consistent depth (mean ± standard deviation (SD), 41.3 ± 2.5 million; range 37.6–45.2 million; *n* = 41). After normalization (variance-stabilized counts for principal component analysis (PCA); DESeq2-normalized counts for differential expression), per-sample median log_2_(count per million (CPM) + 1) values were tightly aligned (Δmedian < 1), indicating no global depth bias (see Supplementary Figure S1A,B). Mapping-rate panels are omitted because unique/multi-mapping logs were not available in the shared dataset. Gene-biotype composition was stable across samples (protein-coding predominated, with modest immunoglobulin loci (IG) and long non-coding RNA (lncRNA) fractions; ribosomal RNA (rRNA) ~1–2% and other classes each <2%), with similar profiles in HP-exposed and unexposed groups.

### 3.2. Mapping Efficiency and Gene Coverage

Trimmed reads were aligned to the human reference genome GRCh38 using HISAT2 [21], and feature-level quantification was carried out with featureCounts [22]. Although explicit alignment summary logs were unavailable, normalized gene expression patterns and mapped gene distributions confirmed high alignment quality.

Group-level sequencing metrics were comparable across tissues. Tumor libraries yielded an average of 42.8 million raw reads per sample with normalized median counts of 23.4 and an estimated rRNA content of 2.2%. NAT libraries averaged 39.2 million raw reads with normalized median counts of 22.9 and rRNA content of 1.9%. These values indicate uniform depth and low rRNA contamination across groups, supporting reliable downstream differential expression and network analyses.

The results for read depth and rRNA content showed clear similarity across the research groups, as the genes per sample ranged between 17,000 and 17,500, which is a reasonable number consistent with the expectations for the tissue transcriptome, ensuring full and appropriate coverage for functional analysis. As for quantification, the gene biotype was used, while for read summarization, GRCh38 was applied as follows: 20,014 coding protein genes, 16,086 lncRNAs, 15,206 pseudogenes. In addition, several smaller RNA categories were represented, such as misc RNA (2219), small nuclear RNA (snRNA) (1910), microRNA (miRNA) (1877), and small nucleolar RNA (snoRNA) (942). Other categories included in these results at this stage were unknown (1026), immunoglobulin loci (IG; 214), T-cell receptor loci (TR; 196), rRNA (53), small Cajal body-specific RNA (scaRNA) (49), mitochondrial tRNA (22), Artifact (19), ribozyme (8), sRNA (7), mitochondrial rRNA (5), vault_RNA (2), and scRNA (1). It was clear that the dominant categories were Protein-coding, lncRNA, and pseudogene, and featureCounts were used to characterize the universe features of all the categories. No rRNA features were considered in the downstream expression analysis and functional analysis.

### 3.3. Transcriptomic Distributions and Principal Component Analysis (PCA)

The global expression patterns were further explored using log_2_-transformed count distributions and dimensionality reduction. Boxplots of normalized gene count confirmed uniform expression across tumor and NAT groups without extreme outliers, reflecting consistent RNA extraction and library preparation—expression distribution quality check. Sample-wise boxplots of log_2_(normalized counts + 1) showed tightly aligned medians and interquartile ranges across all libraries, with only sporadic high-value outliers expected for highly expressed genes. Distributions for tumor and NAT largely overlapped; NAT exhibited a modestly higher central tendency in some samples, but differences were minor after normalization. The uniform spread and absence of systematic shifts indicate effective normalization, comparable library complexity, and minimal batch effects, supporting the validity of subsequent differential expression, enrichment, and network analyses.

**Figure 3:**
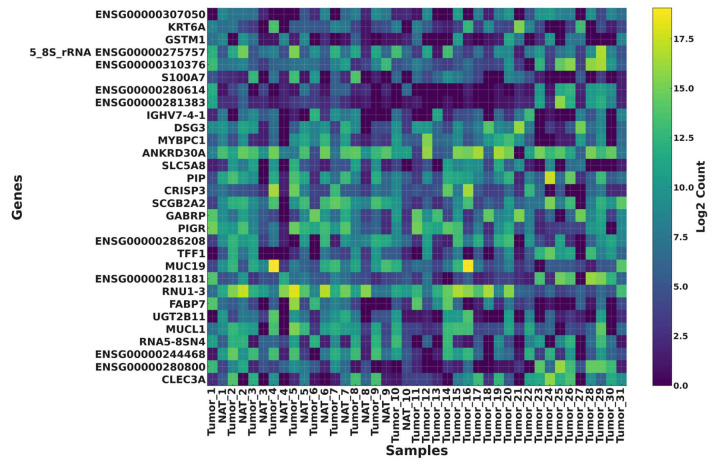
Heatmap of the top 30 most variable genes across tumor and NAT.

For the heatmap, most variable genes were defined as the 30 genes with the highest gene-wise variance across all samples, computed on variance-stabilized expression values (DESeq2) after low-expression filtering. Heatmaps of the top 30 most variable genes in Figure 3 revealed clear sample clustering patterns. Tumor and NAT displayed distinct global signatures, highlighting biologically meaningful differences. The number of detected genes per sample, summarized in Figure 3, reinforced the robustness of transcriptomic coverage across all 41 samples. For the per-sample gene detection, the complexity of the library was quantified as the number of genes with non-zero counts after filtering. It showed a unique unimodal distribution with around 36–37 K features and 31 K to 38.5 K for spanning across samples. The results showed that all the libraries analyzed were perfectly centered around the middle, with only one tail recorded and no abnormal patterns or secondary modes, which clearly indicates the absence of any specific batch structure among the tissues or any of the treatment groups. The data revealed high and distinct uniformity for both protein-coding and non-coding genes in GRCh38, which supported the stability and robustness of downstream differential expression enrichment, functional, and network analysis.

**Figure 4:**
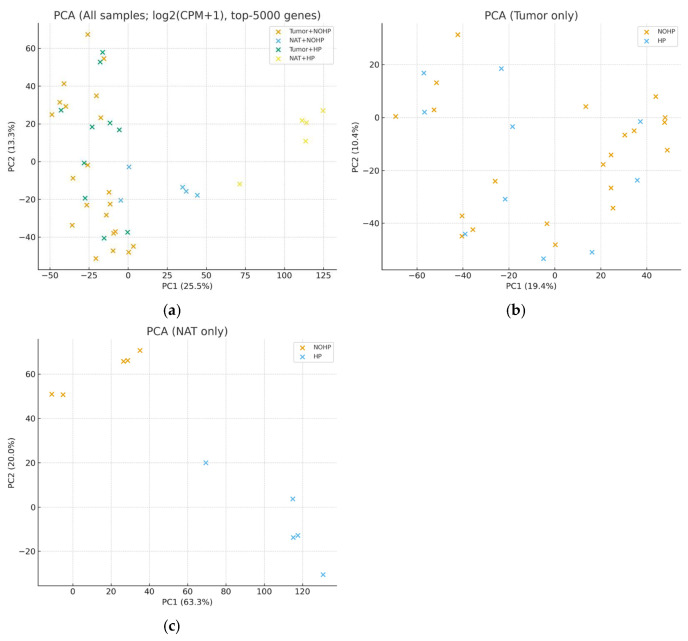
(**a**) All samples. Tissue drives the major axis (PC1 = 25.5%; PC2 = 13.3%). HP’s contribution was quantified on PC2 within tissue: in NAT, HP vs. HP− showed a significant shift; in tumor, the shift was not significant. (**b**) Tumor only. Tumor samples colored by HP status show substantial overlap (panel-specific variance: PC1 = 19.4%, PC2 = 10.4%). (**c**) NAT only. NAT samples colored by HP status show clear separation (panel-specific variance: PC1 = 63.3%, PC2 = 20.0%). Consistent with panel a, the HP shift within NAT is significant. HP = hydroxyprogesterone; Adjacent HP = NAT from HP-treated patients; Adjacent HP− = NAT from untreated patients; Tumor HP = tumor tissue from HP-treated patients; Tumor HP− = tumor tissue from untreated patients.

PCA provided an unsupervised assessment of transcriptomic divergence between groups, as shown in Figure 4. Tumor NATs are separated clearly along the first principal component, reflecting intrinsic differences in malignant and non-malignant transcriptional programs (Figure 4a). HP-treated tumor samples demonstrated a moderate directional shift along the second component, suggesting that HP induces consistent tissue-specific transcriptomic modulation, consistent with prior perioperative hormone modulation studies [15,31] (Figure 4b). PC1 separates Tumor vs. NAT (25.5% of variance); HP exposure separates NAT along PC2 (13.3%), while the HP shift on PC2 is modest and not significant (Figure 4c).

### 3.4. Differential Expression and Functional Insights

**Figure 5:**
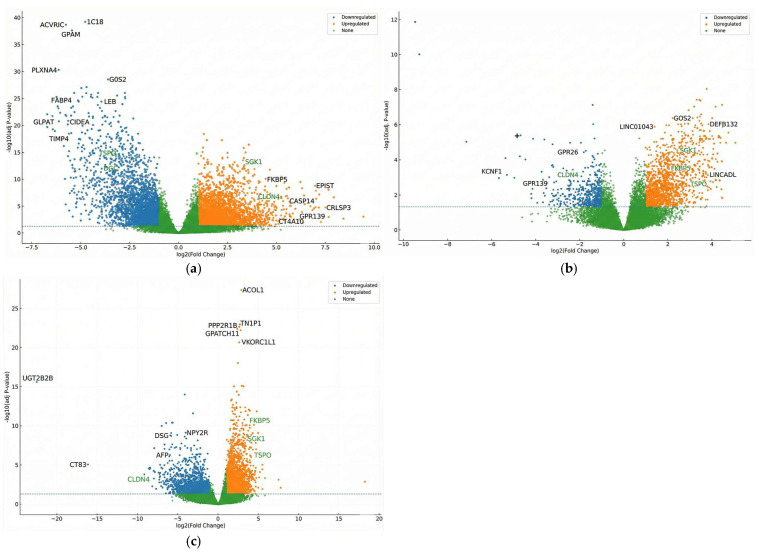
(**a**) Volcano plot comparing tumor and NAT samples. The x-axis represents log_2_ fold change, and the y-axis represents –log_10_ adjusted *p*-value; (**b**) volcano plot showing differential expression between hydroxyprogesterone-treated and untreated tumor samples; (**c**) volcano plot illustrating differential expression in NAT with and without hydroxyprogesterone treatment.

Differential gene expression (DGE) analysis was conducted across tumor versus NAT, HP-treated versus untreated samples, and within each tissue type stratified by treatment. Normalized count matrices generated by DESeq2 and edgeR revealed a clear separation of gene expression profiles, and Figure 5 illustrates the magnitude and significance of transcriptional differences. Significant DEGs: Tumor vs. NAT = 9177 (Figure 5a); Tumor HP+ vs. Tumor HP*−* = 2452 (Figure 5b); NAT HP+ vs. NAT HP− = 5622 (Figure 5c).

The largest differences were between tumor and NAT, characterized by tight-junction/ECM remodeling and metabolic shifts, with NF-κB–linked inflammatory crosstalk present but secondary to these programs. Consistently, CLDN4 was higher in tumors (log_2_FC = +1.873, FDR = 1.43 × 10^−4^) [27]. HP exposure led to moderate but biologically meaningful expression shifts. In adjacent tissue, HP increased FKBP5 (log_2_FC = +2.129, FDR = 0.0158) and other steroid-responsive signals, with enrichment for fatty-acid/xenobiotic/cholesterol gene sets; in tumors, HP effects were smaller, consistent with findings reported in 2018 by Chatterjee et al. [15]. NAT showed the clearest HP-aligned changes, particularly steroid/PPAR–lipid/xenobiotic responses (e.g., FKBP5), consistent with non-canonical progesterone effects [32,33,34].

Functional enrichment analyses using GO, KEGG, and GSEA provided system-level insights into these transcriptional patterns. Tumor samples were enriched in cell cycle, DNA replication, and oncogenic signaling pathways. HP treatment enhanced pathways related to oxidative stress response, inflammatory pathways, and NF-κB-related pathways, steroid biosynthesis, and extracellular matrix remodeling [26,27,35]. These findings support the hypothesis that HP influences both intrinsic tumor programs and the tumor microenvironment.

The scientific framework used in the study indicates two main complementary and important axes in the biology of breast cancer, with clear specificity. The first axis is the HP-responsive steroid/PPAR–lipid–xenobiotic pathway, which appears more prominent and evident in the adjacent tissue, where the pharmacodynamic readout is strong and profound. As for the second axis, the intrinsic pathway of the tumor is driven by epithelial junction and ECM-remodeling signals, which form the basis for invasion and survival [15,34]. What distinguishes this model and the dual perspective for reviewing the data is that it leads to avoiding the single gene tunnel vision and also directs attention to the level of the dual pathways by focusing on the different cellular functional pathways in breast cancer [35].

In this scheme, a strong compartment indicates specific HP engagement [36]. FKBP5 increased with HP in NAT. The corresponding tumor effect was limited and did not remain robust to multiple testing. This is precisely the expected result of asymmetry if the HP is reprogrammed and changed primarily perioperatively, reprograms steroid/PPAR signaling and xenobiotic/lipid handling in non-malignant epithelial or stromal settings [34]. Existing tumors will remain temporarily stabilized within their oncogenic circuitry [15]. In the HP-enriched WGCNA module, FKBP5 has a membership that supports its essential role as an immediate pharmacodynamics readout in place of a downstream spectator. Effectively, FKBP5 represents a concrete and tangible target for validation in mRNA, qPCR, protein, or IHC and patient satisfaction (HP+ vs. HP*−*) studies over time to track when HP’s effects begin and how long they last.

In comparison, CLDN4 represents a primary tumor pathway of epithelial junction remodeling and ECM interaction [37]. In the tumor vs. NAT comparison, the elevations are substantial and statistically robust. CLDN4 is embedded in tight-junction/ECM pathways and is in a tumor-enriched WGCNA neighborhood that aligns with cancer invasion-competent adhesion states. To support and add independent confirmation for CLDN4, we need to perform an independent experiment using qPCR. The function of CLDN4 acts like a bridge or an anchor for a specific phenotype of the malignant epithelial: simply, it combines and integrates with integrin/tetraspanin signaling at the membrane, organizes barrier properties and polarity of the membrane in addition to keeping up with the transcriptional hallmark of the epithelial remodeling that facilitates dissemination [38,39]. Accordingly, while FKBP5 serves as a measure of HP exposure in pre-tumoral tissue, CLDN4 represents the baseline for tumor structure that HP is not expected to alter on its own.

TSPO and SGK1 are considered as exploratory hypothesis candidates to widen the mechanistic study coverage. Both TSPO and SGK1 show steroid/PPAR–lipid/xenobiotic pathway binding tendencies that are clearly consistent and relevant to HP, but statistically, they appear to be modestly significant and more dependent on the biological context. In addition, its biological functions fill the gap left by the primary candidates and are strongly supported by statistics. TSPO links signals responsible for cholesterol transport in mitochondria as well as cellular stress responses, providing a pathway through which metabolic and oxidation–reduction processes can be modulated to alter the nature of cellular motility and sensitivity to treatment; SGK1, a steroid-responsive kinase that biologically emanates from PI3K-AKT, is a potential channel for a wide range of hormonally mediated signals responsible for ion transport and cellular control of junctions [40]. The network diagram of the proteins indicates a regulatory bridge between CLDN4, WNK4, and SGK1, providing a mechanistic pathway from FKBP5 and SGK1 steroid signaling to CLDN4 junction reorganization [41,42,43]. Thus, we conclude that these secondary nodes may help to demonstrate biologically that this complex pathological system functions as a single unit with a highly interconnected system along the lines of steroid → junction/ECM → mitochondrial/ion transport, and not merely as a system based on isolated and noisy genes operating independently without any complex biological system consisting of an interlocking set of interconnected pathways.

It is worth noting that the statistical data indicate a variability in gene sets, especially those related to the subtype. These cellular effects are dependent on the compartment and exposure (e.g., FKBP5↑ in NAT with HP; CLDN4↑ in tumors; TSPO lower in tumors than NAT. This leads us to conclude that some of the identified genes may have a significant and guaranteed relationship within certain strata (ER/PR status, grade, HP exposure timing/dose). The conclusion is that there are two motivations regarding interpretation and design for the future. First, future and upcoming analyses should maintain and emphasize the survival of interaction terms (e.g., tissue × HP, ER status × HP) and avoid any secondary side effects due to genetic subgroups (i.e., predefined stratification variables such as tissue type and ER status, rather than newly identified subgroups) in an exaggerated manner. Second, the validity and strength of hypotheses related to subgroups should be pre-verified instead of relying on clinically related signals.

Together, these findings support a coherent working model. Primary markers—FKBP5 and CLDN4 carry the headline: FKBP5 reports on the HP-engaged steroid/PPAR axis in adjacent tissue, while CLDN4 reports on tumor epithelial remodeling that underlies invasion. Exploratory markers TSPO and SGK1 extend the map to mitochondrial stress and kinase-driven ion-junction control, providing explanatory links between endocrine cues and barrier/ECM phenotypes. This division of labor clarifies why HP’s most visible transcriptomic imprint lies in motility/stress-handling nodes outside the tumor core, whereas the tumor state remains dominated by adhesion and survival pathways.

### 3.5. Co-Expression Modules and Hub Genes (WGCNA)

**Figure 6:**
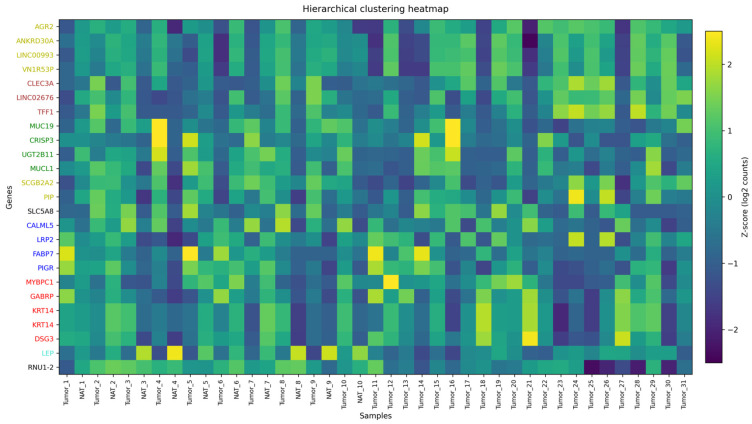
Gene dendrogram and module colors for the Tumor vs. NAT comparison.

**Figure 7:**
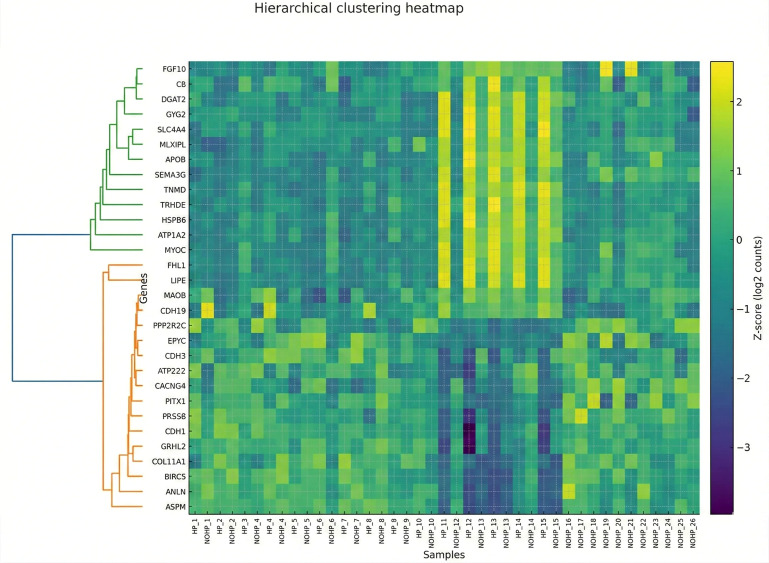
Gene dendrogram and module colors for the HP+ vs. HP− comparison.

WGCNA revealed higher-order gene modules associated with tumor biology and HP treatment. Hierarchical clustering of variance-stabilized expression values identified multiple co-expressed modules, visualized in Figure 6 and Figure 7 and summarized in Table 2 and Table 3. Module–trait correlations. In the Tumor vs. NAT network, the turquoise module (putative Tumor-axis) showed a strong positive association with Tumor (*r* = 0.85, BH-*p* = 1.41 × 10^−11^), whereas the black module (putative NAT-axis) was negatively associated (*r* = −0.70, BH-*p* = 9.46 × 10^−7^). Additional modules had weaker associations (e.g., blue: *r* = −0.37, BH-*p* = 0.039). In the Tumor HP+ vs. Tumor HP*−* network, the top association with HP exposure was observed for the black module (*r* = −0.33, *p* = 0.035, BH-*p* = 0.118), followed by turquoise (*r* = 0.31, *p* = 0.050, BH-*p* = 0.118). Thus, the two principal modules defining the dual-axis model (Tumor-axis = turquoise; NAT-axis = black) are quantitatively supported in Tumor–NAT stratification, while HP effects within tumor are nominal and do not survive multiple-testing correction.

WGCNA found the presence of two hub structures with distinct characteristics. The HP-sensitive module (Hub 1; HP+ vs. HP*−*) is centered on PRKG1 and its direct effectors in cytoskeletal control (VASP, ACTA1, TNNT1) and excitation–contraction/Ca^2+^ handling (PLN), together with the large-conductance Ca^2+^-activated K^+^ channel complex (KCNMA1/KCNMB2/KCNMB4). This configuration shows consistency with the nitric oxide (NO)–soluble guanylyl cyclase (sGC)–cyclic guanosine monophosphate (cGMP)–protein kinase G (PKG) axis, which regulates actin dynamics and cell motility via VASP phosphorylation, while large-conductance Ca2+-activated K+ (BK)-channel activity links ionic/mechanical cues to invasive behavior [44,45]. Peripheral connections (ABCA6, RASGRP3, BARX2) indicate their role in coupling with lipid/xenobiotic transport and Ras–ERK/PI3K signal entry points, confirming that HP can modulate and shift in parallel with the metabolic handling of lipids and stressors [46,47,48].

On the other hand, the tumor-vs-NAT module (Hub 2) encapsulates canonical epithelial breast-cancer programs: steroid signaling and chaperone dependence (ESR1, HSP90AA1) [49], integrin/tetraspanin-organized adhesion and invasion (ITGA5 with CD151/TM4SF5 neighborhood) [25], stemness and therapy tolerance (PROM1/CD133) [50], and transporter/secretome remodeling (ABCC8, PATE-family/LY6 cluster). Considering the two hubs, Hub 2 represents the baseline tumor architecture that sustains proliferation, survival, and dissemination, while Hub 1 reflects an HP-tunable cytoskeleton/ion-channel–lipid module that can modulate motility and stress adaptation.

In the case of integrating both networks together, we conclude that they support a solid and coherent model. Considering the adjacent tissue (and HP-responsive tumor contexts), HP primarily engages a cGMP–PKG–VASP/actin axis [44,45] with BK-channel and lipid-transport crosstalk [51], while at the same time, the cellular biological properties of the tumor remain under the control of ER/HSP90 signaling [49], integrin-ECM remodeling, stemness, and nutrient transport. In this context, the division of labor explains why HP exposure is most visible in motility and stress-handling nodes (Hub 1), while core endocrine and adhesion programs (Hub 2) define the tumor state. The direct readouts of this dual model and the perturbations for validation include, for example, pVASP(Ser239) and cGMP as PKG pharmacodynamic markers in HP+ samples; migration/invasion assays ± PKG or BK-channel modulators; and correlation of ITGA5/CD151, PROM1, and SLC1A5-like transporters with invasive and metabolic phenotypes [38,50,51].

Modules correlated with tumor status were enriched for cell cycle regulation, proteostasis, and epithelial differentiation. Modules linked to HP exposure included genes involved in oxidative stress, extracellular matrix dynamics, and inflammatory pathways, and NF-κB is related [6]. WGCNA highlighted module-level structure rather than generic hubs, a tumor-enriched tight-junction/ECM module containing CLDN4, and an HP-enriched steroid/PPAR–lipid module containing FKBP5 [34]. TSPO and SGK1 showed contextual connections and are treated as exploratory; this pattern aligns with reports that progestins engage steroid-responsive metabolic programs with NF-κB–linked inflammatory cross-talk [15,40]. These co-expression modules highlight the systems-level impact of HP, linking stress adaptation, inflammatory pathways, and NF-κB, which are related, and remodeling to specific hub genes that represent plausible biomarkers or regulatory nodes.

### 3.6. Clinical Implications and Biomarker Potential

HP primarily reprograms adjacent tissue toward a steroid/PPAR–lipid/xenobiotic response (e.g., FKBP5↑), whereas tumor tissue shows tight-junction/ECM remodeling (e.g., CLDN4↑). Practically, FKBP5 is the leading pharmacodynamic candidate for monitoring HP engagement (adjacent/peripheral compartments), CLDN4 informs tumor epithelial remodeling, which should serve as a complementary context readout (e.g., perioperative risk stratification or supportive interpretation of exposure), ideally confirmed at the protein/serum level and interpreted alongside inflammatory markers and clinical covariates.

As a discovery-level, hypothesis-generating re-analysis, these results are intended to prioritize candidate biomarkers and pathway signals for follow-up evaluation in independent datasets and future studies. Group sizes were modest, particularly in NAT (HP+ *n* = 5; HP− *n* = 5) which limits statistical power to detect small-to-moderate HP-associated effects after multiple-testing correction and increases uncertainty in effect-size estimates. In addition, per-sample clinical metadata were incomplete and non-uniform (age, stage, survival outcomes, and ER/PR/HER2 status), introducing potential residual confounding. These constraints are amplified for subtype-stratified or interaction-based analyses. Subtype-stratified interpretations are exploratory given the available metadata and small effective subgroup sizes. Next steps include replication in independent cohorts with standardized clinical annotation, followed by orthogonal confirmation of FKBP5 and CLDN4 in tumor and NAT (e.g., qPCR and immunohistochemistry) and exploratory evaluation of potential circulating readouts, ideally in prospective perioperative studies with covariate-adjusted and subtype-aware analyses.

## 4. Conclusions

This re-analysis study supports a dual-axis model of peri-operative 17-OHPC (HP): a steroid/PPAR–lipid/xenobiotic program most evident in NAT and a tight-junction/ECM-remodeling program dominating the tumor. HP-related shifts are strong in NAT but modest in tumor, consistent with bulk heterogeneity and short pre-operative exposure. These results nominate FKBP5 (exposure readout in NAT) and CLDN4 (tumor context) for orthogonal validation. Overall, these findings support a dual-axis working model and nominate FKBP5 and CLDN4 as candidate biomarkers for follow-up evaluation in independent datasets and future studies.

## Figures and Tables

**Table 1:** Sample grouping details.

Group	Tissue Type	HP Exposure	Sample Count
NAT HP−	NAT	Not exposed	5
NAT HP+	NAT	Exposed	5
Tumor HP−	Tumor	Not exposed	13
Tumor HP+	Tumor	Exposed	18

**Table 2:** WGCNA module summary for tumor vs. NAT.

Module Color	Gene Count	Example Hub Gene
Black	14	MAGEA11
Blue	201	PAX6
Brown	96	ABCC8
Green	43	ITGA5
Red	78	PROM1
Turquoise	258	HSP90AA1
Yellow	82	ESR1

**Table 3:** WGCNA module summary for HP+ vs. HP−.

Module Color	Gene Count	Example Hub Gene
Black	57	BARX2
Blue	187	ACTA1
Brown	101	RASGRP3
Cyan	21	ABCA6
Green	47	PRKG1
Grey	72	SH2D6
Red	78	PROM1
Turquoise	251	KIF5B
Yellow	84	SLC1A5

## Data Availability

The original contributions presented in this study are included in the article. Further inquiries can be directed to the corresponding author.

## Supplementary Materials

The following supporting information can be downloaded at https://www.mdpi.com/article/10.3390/cimb48010108/s1.

## Abbreviations

The following abbreviations are used in this manuscript:

17-OHPC	17α-hydroxyprogesterone caproate
BP	Biological Process (Gene Ontology)
CC	Cellular Component (Gene Ontology)
DEG	Differentially Expressed Gene
ER	Estrogen Receptor
ER stress	Endoplasmic Reticulum Stress
FC	Fold Change
FDR	False Discovery Rate
GO	Gene Ontology
GRCh38	Genome Reference Consortium Human Build 38
GSEA	Gene Set Enrichment Analysis
HP	Hydroxyprogesterone
HP+	Hydroxyprogesterone-treated
HP−	Hydroxyprogesterone-untreated
Log_2_FC	Log_2_ Fold Change
MF	Molecular Function (Gene Ontology)
NAT	Normal Adjacent Tissue
NOHP	No Hydroxyprogesterone
ORA	Overrepresentation Analysis
PR	Progesterone Receptor
QC	Quality Control
RNA-seq	RNA Sequencing
rRNA	Ribosomal RNA
TMM	Trimmed Mean of M-values
WGCNA	Weighted Gene Co-expression Network Analysis
