# Supplementary material for: Comprehensive Transcriptomic Analysis and Biomarker Prioritization of Hydroxyprogesterone in Breast Cancer
Source: Curr Issues Mol Biol. 2026 Jan 20;48(1):108. doi: 10.3390/cimb48010108 (PMC12839910; doi:10.3390/cimb48010108)
Supplement: Supplementary file 1 [file cimb-48-00108-s001.zip › cimb-4060860-supplementary.pdf]

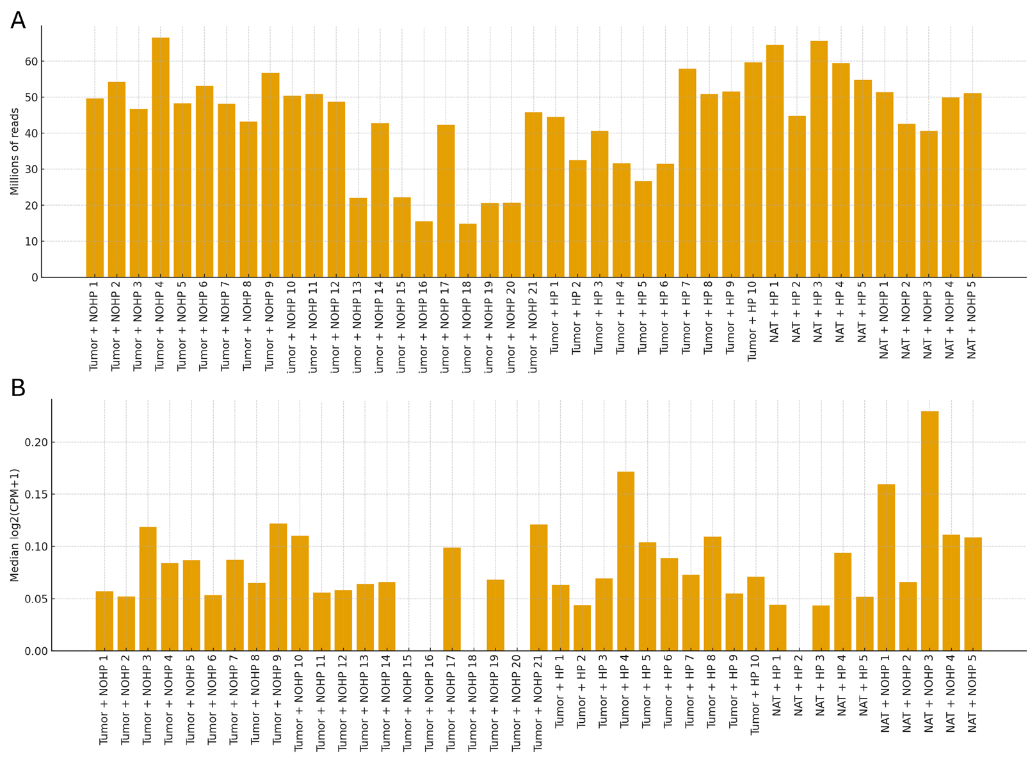

Figure S1. Library quality control. (A) Total gene-assigned reads per sample (millions), enumerated by group. (B) Per-sample median $\log_2$(CPM+1) after normalization**.**
